# 6-Benzyl­oxycoumarin

**DOI:** 10.1107/S1600536810029430

**Published:** 2010-07-31

**Authors:** Morina Adfa, Mamoru Koketsu, Masahiro Ebihara

**Affiliations:** a Department of Materials Science and Technology, Faculty of Engineering, Gifu University, 1-1 Yanagido, Gifu 501-1193, Japan; b Department of Chemistry, Faculty of Engineering, Gifu University, 1-1 Yanagido, Gifu 501-1193, Japan

## Abstract

In the title compound, 6-benz­yloxy-2*H*-1-benzopyran-2-one, C_16_H_12_O_3_, the coumarin unit and benzyl plane in the mol­ecule are perpendicular to each other [86.92 (7)°]. The crystal packing is stabilized by π–π stacking inter­actions, with an inter­planar separation between inversion-related coumarin units of 3.618 (3) Å. The crystal structure shows inter­molecular C—H⋯O hydrogen bonding between neighboring mol­ecules.

## Related literature

For general background to coumarin, see: Adfa *et al.* (2010 ▶); Gunnewegh *et al.* (1995 ▶); Li *et al.* (1998 ▶); Murray *et al.* (1982 ▶); Schönberg & Latif (1954 ▶). For related compounds, see: Chinnakali *et al.* (1998 ▶); Jasinski *et al.* (2003 ▶).
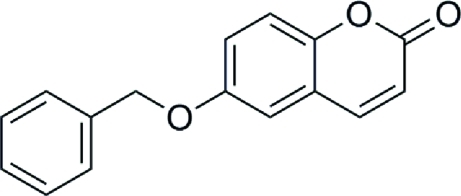


## Experimental

### 

#### Crystal data

C_16_H_12_O_3_
*M* *_r_* = 252.26
Monoclinic, 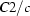

*a* = 20.391 (12) Å
*b* = 6.732 (4) Å
*c* = 18.844 (11) Å
β = 94.833 (8)°
*V* = 2578 (3) Å^3^
*Z* = 8
Mo *K*α radiation
μ = 0.09 mm^−1^
*T* = 296 K
0.30 × 0.10 × 0.10 mm

#### Data collection

Rigaku AFC7R Mercury CCD diffractometer
10197 measured reflections
2912 independent reflections
2070 reflections with *I* > 2σ(*I*)
*R*_int_ = 0.039

#### Refinement

*R*[*F* ^2^ > 2σ(*F* ^2^)] = 0.075
*wR*(*F* ^2^) = 0.190
*S* = 1.20
2912 reflections
172 parameters
H-atom parameters constrained
Δρ_max_ = 0.16 e Å^−3^
Δρ_min_ = −0.18 e Å^−3^

### 

Data collection: *CrystalClear* (Rigaku/MSC, 2001 ▶); cell refinement: *CrystalClear*; data reduction: *Yadokari-XG 2009* (Wakita, 2001 ▶; Kabuto *et al.*, 2009 ▶); program(s) used to solve structure: *SIR97* (Altomare *et al.*, 1999 ▶); program(s) used to refine structure: *SHELXL97* (Sheldrick, 2008 ▶); molecular graphics: *Yadokari-XG 2009* and *Mercury* (Macrae *et al.*, 2006 ▶); software used to prepare material for publication: *publCIF* (Westrip, 2010 ▶).

## Supplementary Material

Crystal structure: contains datablocks I, General. DOI: 10.1107/S1600536810029430/zl2289sup1.cif

Structure factors: contains datablocks I. DOI: 10.1107/S1600536810029430/zl2289Isup2.hkl

Additional supplementary materials:  crystallographic information; 3D view; checkCIF report

## Figures and Tables

**Table 1:** Hydrogen-bond geometry (Å, °)

*D*—H⋯*A*	*D*—H	H⋯*A*	*D*⋯*A*	*D*—H⋯*A*
C2—H2⋯O2^i^	0.93	2.62	3.472 (3)	153
C3—H3⋯O2^ii^	0.93	2.58	3.461 (3)	159
C5—H5⋯O1^ii^	0.93	2.59	3.501 (3)	168
C8—H8⋯O3^iii^	0.93	2.54	3.460 (3)	170
C16—H16⋯O2^iv^	0.93	2.56	3.421 (3)	154

Symmetry codes: (i) 
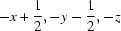
; (ii) 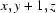; (iii) 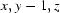; (iv) 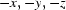.

**Fig. 1.**
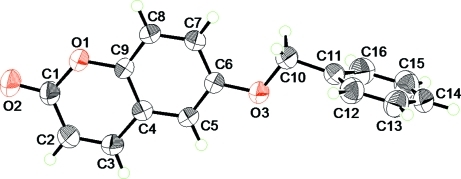
The molecular structure and atom-numbering scheme of the title compound. Displacement ellipsoids are drawn at the 30% probability level and H atoms are shown as small spheres of arbitrary radii.

**Fig. 2.**
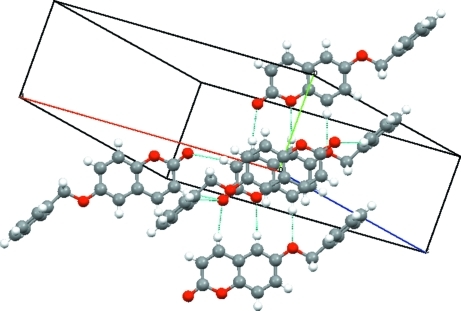
Packing diagram of the title compound viewed perpendicular to the coumarin plane. The C—H···O hydrogen bonding interactions between the neighboring molecules are shown as dashed lines.

## supplementary crystallographic information

### Comment

Coumarin and its derivatives have been found to exhibit various biological and pharmacological activities, such as molluscicidal (Schönberg and Latif, 1954), termiticidal (Adfa *et al.*, 2010), rodenticidal, anthelmintic, antibacterial, antioxidant, anti-inflammatory, and anti-cancer, and they have been used as anticoagulant agents and fluorescent brighteners (Murray *et al.*, 1982; Gunnewegh *et al.*, 1995; Li *et al.*, 1998). The title compound is one of the derivatives of coumarin. In order to investigate the structure activity relationship (SAR) of the compound for biological activities, it is essential to determine the configuration of 6-benzyloxycoumarin.

The molecular structure of the title compound is illustrated in Fig. 1. The coumarin moiety and benzyl planes (r.m.s deviations 0.039 and 0.017 Å) in the molecule are perpendicular to each other with a dihedral angle between the plane of the atoms O1–O3, C1–C9 and that of C10–C15 of 86.92 (7)°. The structure shows intermolecular C—H···O hydrogen bonding between four neighboring molecules (Table 1 and Fig. 2): C3···O2^i^, C5···O1^i^ and C8^i^···O3 [symmetry code (i) *x*, 1 + *y*, *z*]; C3^ii^···O2, C5^ii^···O1 and C8···O3^ii^ [symmetry code (ii) *x*, -1 + *y*, *z*]; C2···O2^iii^ and C2^iii^···O2 [symmetry code (iii) 1/2 - *x*, -1/2 - *y*, -*z*]; C16···O2^iv^ and C16^iv^···O2 [symmetry code (iv) -*x*, -*y*, -*z*]. There also exist π–π stacking interactions between the coumarin moieties with an interplanar separation of 3.618 (3) Å (based on all atoms but the phenyl ring C atoms, symmetry operator for the second molecule iv). Similar structural features are also observed in other coumarin derivatives (Chinnakali *et al.*, 1998; Jasinski *et al.*, 2003).

### Experimental

A mixture of 6-hydroxycoumarin (30 mg, 0.19 mmol), benzyl bromide (43.8 cm^3^, 0.37 mmol), and potassium carbonate (51 mg, 0.37 mmol) in DMF (5.0 cm^3^) was stirred at 353 K for 1.5 h. The reaction mixture was extracted with ethyl acetate and washed with water. The organic layer was dried over sodium sulfate and evaporated to dryness. The residue was purified by column chromatography on silica gel with n-hexane/ethyl acetate (7:3) to give the title compound (40.3 mg, 86.4%) as colourless crystals, m.p. 385 K. ^1^H-NMR (600 MHz, CDCl_3_): δ 5.10 (2*H*, *s*, CH_2_), 6.42 (1*H*, *d*, *J* = 9.6 Hz), 6.99 (1*H*, *d*, *J* = 2.8 Hz), 7.18 (1*H*, *dd*, *J* = 8.9 and 2.8 Hz), 7.26 (1*H*, *d*, *J* = 8.9 Hz), 7.34–7.44 (5*H*, *m*, Ar), 7.63 (1*H*, *d*, *J* = 9.6 Hz); ^13^C-NMR (150 MHz, CDCl_3_): δ 70.8, 111.5, 117.2, 118.1, 119.3, 120.3, 127.6, 128.4, 128.8, 136.4, 143.3, 148.7, 155.3, 161.1. Single crystals of 6-benzyloxycoumarin were grown by recrystallization from a solution in chloroform-hexane (10:3).

### Refinement

C-bound H atoms were placed in idealized positions and treated as riding atoms with C—H distances in the range 0.93–0.97 Å and with *U*_iso_(H) = 1.2*U*_eq_(C) for the H atoms.

### Crystal data

**Table:**

C_16_H_12_O_3_	*F*(000) = 1056
*M**_r_* = 252.26	*D*_x_ = 1.300 Mg m^−^^3^
Monoclinic, *C*2/*c*	Melting point: 385 K
Hall symbol: -C 2yc	Mo *K*α radiation, λ = 0.71070 Å
*a* = 20.391 (12) Å	Cell parameters from 2417 reflections
*b* = 6.732 (4) Å	θ = 3.1–27.5°
*c* = 18.844 (11) Å	µ = 0.09 mm^−^^1^
β = 94.833 (8)°	*T* = 296 K
*V* = 2578 (3) Å^3^	Prism, colourless
*Z* = 8	0.30 × 0.10 × 0.10 mm

### Data collection

**Table:**

Rigaku AFC7R Mercury CCD diffractometer	2070 reflections with *I* > 2σ(*I*)
Radiation source: Rotating Anode	*R*_int_ = 0.039
graphite	θ_max_ = 27.5°, θ_min_ = 3.1°
Detector resolution: 14.6199 pixels mm^-1^	*h* = −23→26
dtintegrate.ref scans	*k* = −7→8
10197 measured reflections	*l* = −24→24
2912 independent reflections	

### Refinement

**Table:**

Refinement on *F*^2^	Primary atom site location: structure-invariant direct methods
Least-squares matrix: full	Secondary atom site location: difference Fourier map
*R*[*F*^2^ > 2σ(*F*^2^)] = 0.075	Hydrogen site location: inferred from neighbouring sites
*wR*(*F*^2^) = 0.190	H-atom parameters constrained
*S* = 1.20	*w* = 1/[σ^2^(*F*_o_^2^) + (0.0813*P*)^2^ + 0.4589*P*] where *P* = (*F*_o_^2^ + 2*F*_c_^2^)/3
2912 reflections	(Δ/σ)_max_ < 0.001
172 parameters	Δρ_max_ = 0.16 e Å^−^^3^
0 restraints	Δρ_min_ = −0.18 e Å^−^^3^

### Special details

**Table:**

Geometry. All e.s.d.'s (except the e.s.d. in the dihedral angle between two l.s. planes) are estimated using the full covariance matrix. The cell e.s.d.'s are taken into account individually in the estimation of e.s.d.'s in distances, angles and torsion angles; correlations between e.s.d.'s in cell parameters are only used when they are defined by crystal symmetry. An approximate (isotropic) treatment of cell e.s.d.'s is used for estimating e.s.d.'s involving l.s. planes.
Refinement. Refinement of *F*^2^ against ALL reflections. The weighted *R*-factor *wR* and goodness of fit *S* are based on *F*^2^, conventional *R*-factors *R* are based on *F*, with *F* set to zero for negative *F*^2^. The threshold expression of *F*^2^ > σ(*F*^2^) is used only for calculating *R*-factors(gt) *etc*. and is not relevant to the choice of reflections for refinement. *R*-factors based on *F*^2^ are statistically about twice as large as those based on *F*, and *R*- factors based on ALL data will be even larger.

### Fractional atomic coordinates and isotropic or equivalent isotropic displacement parameters (Å^2^)

**Table:**

	*x*	*y*	*z*	*U*_iso_*/*U*_eq_	
O1	0.08714 (7)	−0.28238 (19)	0.06726 (8)	0.0517 (4)	
C1	0.14413 (11)	−0.2635 (3)	0.03418 (12)	0.0499 (5)	
O2	0.17437 (9)	−0.4146 (2)	0.02481 (10)	0.0698 (5)	
C2	0.16227 (12)	−0.0660 (3)	0.01380 (13)	0.0565 (6)	
H2	0.1997	−0.0486	−0.0105	0.068*	
C3	0.12678 (11)	0.0923 (3)	0.02892 (13)	0.0545 (6)	
H3	0.1395	0.2176	0.0145	0.065*	
C4	0.06915 (10)	0.0725 (3)	0.06722 (11)	0.0441 (5)	
C5	0.03075 (11)	0.2315 (3)	0.08766 (12)	0.0499 (6)	
H5	0.0418	0.3605	0.0757	0.060*	
C6	−0.02290 (11)	0.1989 (3)	0.12518 (13)	0.0505 (5)	
C7	−0.04011 (11)	0.0050 (3)	0.14279 (13)	0.0586 (6)	
H7	−0.0765	−0.0173	0.1683	0.070*	
C8	−0.00300 (11)	−0.1532 (3)	0.12231 (13)	0.0552 (6)	
H8	−0.0144	−0.2823	0.1338	0.066*	
C9	0.05060 (10)	−0.1191 (3)	0.08502 (11)	0.0438 (5)	
O3	−0.05721 (8)	0.3649 (2)	0.14299 (11)	0.0687 (5)	
C10	−0.11309 (13)	0.3365 (3)	0.18242 (16)	0.0676 (7)	
H10A	−0.0995	0.2785	0.2284	0.081*	
H10B	−0.1438	0.2461	0.1570	0.081*	
C11	−0.14576 (11)	0.5327 (3)	0.19239 (12)	0.0512 (6)	
C12	−0.13279 (13)	0.6408 (3)	0.25384 (13)	0.0612 (6)	
H12	−0.1017	0.5947	0.2889	0.073*	
C13	−0.16522 (14)	0.8164 (3)	0.26425 (14)	0.0652 (7)	
H13	−0.1561	0.8872	0.3063	0.078*	
C14	−0.21066 (13)	0.8871 (3)	0.21321 (14)	0.0609 (7)	
H14	−0.2326	1.0056	0.2204	0.073*	
C15	−0.22378 (13)	0.7825 (4)	0.15136 (14)	0.0676 (7)	
H15	−0.2546	0.8303	0.1163	0.081*	
C16	−0.19132 (14)	0.6060 (3)	0.14083 (13)	0.0633 (7)	
H16	−0.2003	0.5361	0.0986	0.076*	

### Atomic displacement parameters (Å^2^)

**Table:**

	*U*^11^	*U*^22^	*U*^33^	*U*^12^	*U*^13^	*U*^23^
O1	0.0507 (9)	0.0364 (7)	0.0700 (10)	0.0055 (6)	0.0165 (7)	0.0010 (6)
C1	0.0495 (13)	0.0468 (11)	0.0544 (13)	0.0074 (9)	0.0111 (10)	−0.0030 (9)
O2	0.0723 (12)	0.0497 (9)	0.0911 (13)	0.0141 (8)	0.0283 (10)	−0.0049 (8)
C2	0.0532 (14)	0.0518 (12)	0.0673 (15)	0.0009 (10)	0.0221 (11)	0.0011 (10)
C3	0.0528 (14)	0.0423 (11)	0.0710 (15)	−0.0025 (9)	0.0213 (11)	0.0034 (10)
C4	0.0417 (12)	0.0379 (10)	0.0530 (12)	−0.0008 (8)	0.0064 (9)	0.0019 (8)
C5	0.0471 (13)	0.0324 (9)	0.0716 (15)	−0.0010 (8)	0.0139 (11)	0.0032 (9)
C6	0.0444 (12)	0.0366 (10)	0.0719 (15)	0.0018 (8)	0.0132 (10)	−0.0017 (9)
C7	0.0472 (13)	0.0428 (11)	0.0891 (17)	0.0001 (9)	0.0253 (12)	0.0079 (11)
C8	0.0497 (13)	0.0350 (10)	0.0832 (16)	−0.0009 (8)	0.0201 (11)	0.0081 (10)
C9	0.0428 (12)	0.0329 (9)	0.0559 (12)	0.0027 (8)	0.0062 (9)	0.0009 (8)
O3	0.0594 (11)	0.0386 (8)	0.1138 (14)	0.0039 (7)	0.0417 (10)	0.0007 (8)
C10	0.0616 (16)	0.0473 (12)	0.099 (2)	0.0020 (11)	0.0363 (14)	0.0014 (12)
C11	0.0481 (13)	0.0437 (11)	0.0648 (14)	0.0003 (9)	0.0223 (11)	−0.0001 (10)
C12	0.0580 (15)	0.0583 (13)	0.0663 (16)	0.0040 (11)	−0.0015 (12)	−0.0023 (11)
C13	0.0728 (18)	0.0573 (13)	0.0664 (15)	0.0008 (12)	0.0112 (13)	−0.0144 (12)
C14	0.0550 (15)	0.0485 (12)	0.0829 (18)	0.0063 (10)	0.0280 (13)	0.0006 (11)
C15	0.0596 (16)	0.0721 (16)	0.0709 (17)	0.0104 (12)	0.0045 (13)	0.0135 (13)
C16	0.0697 (17)	0.0645 (14)	0.0568 (14)	0.0001 (12)	0.0115 (12)	−0.0070 (11)

### Geometric parameters (Å, °)

**Table:**

O1—C1	1.370 (3)	C8—H8	0.9300
O1—C9	1.385 (2)	O3—C10	1.425 (3)
C1—O2	1.210 (2)	C10—C11	1.499 (3)
C1—C2	1.440 (3)	C10—H10A	0.9700
C2—C3	1.333 (3)	C10—H10B	0.9700
C2—H2	0.9300	C11—C12	1.374 (3)
C3—C4	1.436 (3)	C11—C16	1.378 (3)
C3—H3	0.9300	C12—C13	1.377 (3)
C4—C9	1.393 (3)	C12—H12	0.9300
C4—C5	1.399 (3)	C13—C14	1.364 (4)
C5—C6	1.369 (3)	C13—H13	0.9300
C5—H5	0.9300	C14—C15	1.369 (4)
C6—O3	1.375 (2)	C14—H14	0.9300
C6—C7	1.399 (3)	C15—C16	1.382 (3)
C7—C8	1.380 (3)	C15—H15	0.9300
C7—H7	0.9300	C16—H16	0.9300
C8—C9	1.368 (3)		
			
C1—O1—C9	122.04 (15)	O1—C9—C4	120.94 (18)
O2—C1—O1	116.78 (18)	C6—O3—C10	117.63 (16)
O2—C1—C2	126.2 (2)	O3—C10—C11	109.29 (17)
O1—C1—C2	116.97 (17)	O3—C10—H10A	109.8
C3—C2—C1	121.7 (2)	C11—C10—H10A	109.8
C3—C2—H2	119.2	O3—C10—H10B	109.8
C1—C2—H2	119.2	C11—C10—H10B	109.8
C2—C3—C4	121.00 (18)	H10A—C10—H10B	108.3
C2—C3—H3	119.5	C12—C11—C16	118.4 (2)
C4—C3—H3	119.5	C12—C11—C10	121.1 (2)
C9—C4—C5	118.17 (19)	C16—C11—C10	120.5 (2)
C9—C4—C3	117.23 (17)	C11—C12—C13	120.9 (2)
C5—C4—C3	124.61 (18)	C11—C12—H12	119.5
C6—C5—C4	120.65 (18)	C13—C12—H12	119.5
C6—C5—H5	119.7	C14—C13—C12	120.3 (2)
C4—C5—H5	119.7	C14—C13—H13	119.8
C5—C6—O3	116.17 (17)	C12—C13—H13	119.8
C5—C6—C7	119.94 (19)	C13—C14—C15	119.6 (2)
O3—C6—C7	123.9 (2)	C13—C14—H14	120.2
C8—C7—C6	119.9 (2)	C15—C14—H14	120.2
C8—C7—H7	120.0	C14—C15—C16	120.2 (2)
C6—C7—H7	120.0	C14—C15—H15	119.9
C9—C8—C7	119.67 (18)	C16—C15—H15	119.9
C9—C8—H8	120.2	C11—C16—C15	120.6 (2)
C7—C8—H8	120.2	C11—C16—H16	119.7
C8—C9—O1	117.41 (16)	C15—C16—H16	119.7
C8—C9—C4	121.62 (18)		
			
C9—O1—C1—O2	175.73 (19)	C5—C4—C9—C8	−0.9 (3)
C9—O1—C1—C2	−4.4 (3)	C3—C4—C9—C8	179.1 (2)
O2—C1—C2—C3	−177.5 (3)	C5—C4—C9—O1	−179.01 (18)
O1—C1—C2—C3	2.7 (3)	C3—C4—C9—O1	1.0 (3)
C1—C2—C3—C4	0.8 (4)	C5—C6—O3—C10	−179.6 (2)
C2—C3—C4—C9	−2.7 (3)	C7—C6—O3—C10	0.7 (4)
C2—C3—C4—C5	177.3 (2)	C6—O3—C10—C11	−176.5 (2)
C9—C4—C5—C6	1.1 (3)	O3—C10—C11—C12	−96.5 (3)
C3—C4—C5—C6	−178.9 (2)	O3—C10—C11—C16	85.6 (3)
C4—C5—C6—O3	179.72 (19)	C16—C11—C12—C13	1.0 (4)
C4—C5—C6—C7	−0.6 (4)	C10—C11—C12—C13	−176.9 (2)
C5—C6—C7—C8	0.0 (4)	C11—C12—C13—C14	−0.5 (4)
O3—C6—C7—C8	179.6 (2)	C12—C13—C14—C15	−0.2 (4)
C6—C7—C8—C9	0.2 (4)	C13—C14—C15—C16	0.2 (4)
C7—C8—C9—O1	178.4 (2)	C12—C11—C16—C15	−1.0 (4)
C7—C8—C9—C4	0.3 (4)	C10—C11—C16—C15	177.0 (2)
C1—O1—C9—C8	−175.6 (2)	C14—C15—C16—C11	0.3 (4)
C1—O1—C9—C4	2.6 (3)		

### Hydrogen-bond geometry (Å, °)

**Table:**

*D*—H···*A*	*D*—H	H···*A*	*D*···*A*	*D*—H···*A*
C2—H2···O2^i^	0.93	2.62	3.472 (3)	153
C3—H3···O2^ii^	0.93	2.58	3.461 (3)	159
C5—H5···O1^ii^	0.93	2.59	3.501 (3)	168
C8—H8···O3^iii^	0.93	2.54	3.460 (3)	170
C16—H16···O2^iv^	0.93	2.56	3.421 (3)	154

Symmetry codes: (i) −*x*+1/2, −*y*−1/2, −*z*; (ii) *x*, *y*+1, *z*; (iii) *x*, *y*−1, *z*; (iv) −*x*, −*y*, −*z*.

## References

[bb1] Adfa, M., Yoshimura, T., Komura, K. & Koketsu, M. (2010). *J. Chem. Ecol.* **36**, 720–726. 10.1007/s10886-010-9807-1 20563628

[bb2] Altomare, A., Burla, M. C., Camalli, M., Cascarano, G. L., Giacovazzo, C., Guagliardi, A., Moliterni, A. G. G., Polidori, G. & Spagna, R. (1999). *J. Appl. Cryst.* **32**, 115–119.

[bb3] Chinnakali, K., Fun, H.-K., Sriraghavan, K. & Ramakrishnan, V. T. (1998). *Acta Cryst.* C**54**, 542–544. 10.1107/s0108270198017429 10777896

[bb4] Gunnewegh, E. A., Hoefnagel, A. J. & van Bekkum, H. (1995). *J. Mol. Catal. A Chem.* **100**, 87–92.

[bb5] Jasinski, J. P., Jasinski, J. M., Li, Y. & Crosby, D. J. (2003). *Acta Cryst.* E**59**, o153–o154.

[bb6] Kabuto, C., Akine, S., Nemoto, T. & Kwon, E. (2009). *J. Cryst. Soc. Jpn*, **51**, 218–224.

[bb7] Li, T.-S., Zhang, Z.-H., Yang, F. & Fu, C.-G. (1998). *J. Chem. Res. (S)*, pp. 38–39.

[bb8] Macrae, C. F., Edgington, P. R., McCabe, P., Pidcock, E., Shields, G. P., Taylor, R., Towler, M. & van de Streek, J. (2006). *J. Appl. Cryst.* **39**, 453–457.

[bb9] Murray, R. D. H., Mendez, J. & Brown, S. A. (1982). *The Natural Coumarins (Occurrence, Chemistry and Biochemistry)* New York*: *John Wiley & Sons.

[bb10] Rigaku/MSC (2001). *CrystalClear* Rigaku/MSC, The Woodlands, Texas, USA.

[bb11] Schönberg, A. & Latif, N. (1954). *J. Am. Chem. Soc.* **76**, 6208–6210.

[bb12] Sheldrick, G. M. (2008). *Acta Cryst.* A**64**, 112–122. 10.1107/S0108767307043930 18156677

[bb13] Wakita, K. (2001). *Yadokari-XG* Department of Chemistry, Graduate School of Science, The University of Tokyo, Japan.

[bb14] Westrip, S. P. (2010). *J. Appl. Cryst.* **43**, 920–925.

